# Exploring the Landscape of General Surgery in the Adolescent Age Group: Challenges and Considerations

**DOI:** 10.7759/cureus.55754

**Published:** 2024-03-07

**Authors:** Abhishek K Saw, Krishna Murari, Zenith Kerketta, Khushboo Rani, Kritika Srivastava, Nusrat Noor

**Affiliations:** 1 General Surgery, Rajendra Institute of Medical Sciences, Ranchi, IND; 2 Obstetrics and Gynecology, Rajendra Institute of Medical Sciences, Ranchi, IND; 3 General Practice, Clinica Cure Hospital, Ranchi, IND

**Keywords:** intestinal obstruction, intestinal perforation, elective surgery, emergency surgery, adolescent age group

## Abstract

Introduction: The adolescent age group typically ranges from 10 to 19 years. This age group differs from the paediatric and adult populations based on their physiological, psychological, and social behaviour. Patients of this age group usually present with trauma, swellings, burns, hernias, hydroceles, haemorrhoids, fibroadenomas, abscesses, pilonidal diseases, etc. The objective of this study was to identify various causes requiring surgical intervention in adolescent patients and to determine the demography of these patients, reasons for surgery, and surgical outcomes in the patients of the adolescent age group.

Materials and methods: This single-centre, hospital record-based, retrospective, cross-sectional study was conducted on 445 adolescent patients who underwent various general surgical interventions from August 2022 to July 2023 in the Department of General Surgery, Rajendra Institute of Medical Sciences (RIMS), Ranchi.

Results: A total of 445 patients were included in this study; among them, 277 underwent elective surgeries and 168 emergency surgeries. Major surgeries included 315 patients, while 130 were daycare procedures. Males were 294, and 151 were females. Cyst excision was the most performed, followed by fibroadenoma excision. Burn (10.78%) was the most common cause requiring major intervention, followed by intestinal obstruction (6.96%) and perforation (6.51%). Mortality was observed in 6.51% of patients.

Conclusion: In this study, the adolescent age group required more elective surgical care as compared to emergency care. Among major surgeries, abdominal laparotomy was most common, and in daycare procedures as well as overall, cyst excision was most performed.

## Introduction

The domain of general surgery has witnessed transformative changes, driven by innovative techniques, cutting-edge technologies, and a deeper understanding of surgical procedures. While surgical interventions have traditionally been associated with adults, a unique subset of patients exists who demand specific attention and consideration: adolescents. The adolescent age group, typically ranging from 10 to 19 years, presents distinct physiological, psychological, and social characteristics, differentiating them from both paediatric and adult populations [1]. It refers to the period marking the transition from childhood to adulthood [2]. As such, addressing surgical needs in this age group requires a nuanced approach that recognises the challenges and complexities inherent to this transitional phase of life.

The landscape of surgical care for adolescents presents a critical challenge in global healthcare systems, marked by disparities in access and a paucity of comprehensive research. In 2017, an estimated 1.7 billion children and adolescents worldwide were noted to lack access to necessary surgical interventions, particularly in regions grappling with limited resources [3].

Adolescents seek surgical opinions for several conditions, including haemorrhoids, rectal prolapse, pilonidal diseases, nail problems, and keloids [4]. Elective surgeries done in the adolescent age group are less common (14.2%) compared to adults [5], which are commonly for cosmetic correction [6] and bariatric surgeries [7].

Adolescents account for a substantial number of emergency department visits. The Nationwide Inpatient Sample (2003-2011, by the American Association for Surgery of Trauma) [8] analysed the factors influencing mortality and morbidity in emergency general surgeries (EGS). This study reported that the least involved age group was 16-25 years old (6.01%). A lesser incidence of EGS in the young age group leads to less attention on focused research, which leads to a scarcity of literature on this age group [8]. In a study by Ziv et al. (1994), adolescents comprised 15.8% of emergency department visits. Injury-related visits were more common among adolescents (28.6%) than children (23.1%) or adults (18.2%) [9].

Most of the ICU (both adult and paediatric) admissions for the adolescent age group are following elective surgery. Excluding this, trauma is the most common reason for adult ICU admissions. Mortality ranges from 3.4% to 5.2% [10].

The objective of this study was to identify surgical causes requiring surgical intervention in adolescent patients at a tertiary care centre. The study aimed to determine the reasons for surgery and assess surgical outcomes in these patients.

## Materials and methods

This cross-sectional study was conducted within the Department of General Surgery at Rajendra Institute of Medical Sciences (RIMS), Ranchi, a tertiary care centre. The study encompassed the medical records of patients who had undergone general surgeries over a one-year span, from August 2022 to July 2023. These records were sourced from the Medical Record Department files.

Inclusion criteria include patients of either sex, aged 13 to 19 years, who underwent either elective or emergency surgeries for both major and minor procedures at RIMS, Ranchi. The study excluded adolescents who received medical treatment and those who underwent minor surgeries (such as basic wound suturing and dressing), re-exploring surgeries, and primary and secondary suturing. Information concerning the demographic particulars, clinical presentation, diagnostic procedures, surgical interventions, and hospital stays of the adolescent patients was extracted from the medical records. Data encompassing management approaches and surgical outcomes were also documented. The information extracted from the records was meticulously entered into a pre-designed and pilot-tested form.

The compiled data was inputted into Microsoft Excel spreadsheets (Microsoft Corporation, Washington, USA), and the SPSS Statistics version 22.0 (IBM Corp. Released 2013. IBM SPSS Statistics for Windows, Version 22.0. Armonk, NY: IBM Corp.) was employed for the analysis, in which a two-sided p-value of 0.05 was recognised as statistically significant. For continuous variables with a normal distribution, their representation consisted of the mean along with the standard deviation. Categorical variables were depicted through frequencies. To establish significance, qualitative comparisons were assessed using the chi-square test or Fisher’s exact test for data calculations involving p-values. Quantitative variables were evaluated using the student’s t-test.

## Results

**Figure 1:**
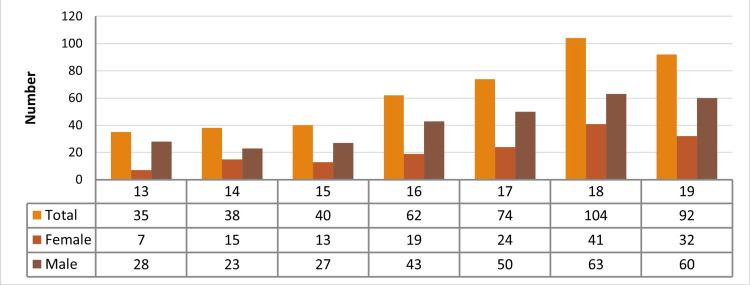
Descriptive analysis of age and sex

In a one-year span, 5699 surgical procedures were done, out of which 4148 were elective and 1551 were emergency cases. Out of 5699 total surgeries performed in one year, 445 were in the adolescent age group, and the incidence was 7.80%. There were 294 (66.07%) males and 151 (33.93%) females in the population studied (Figure 1).

**Figure 2:**
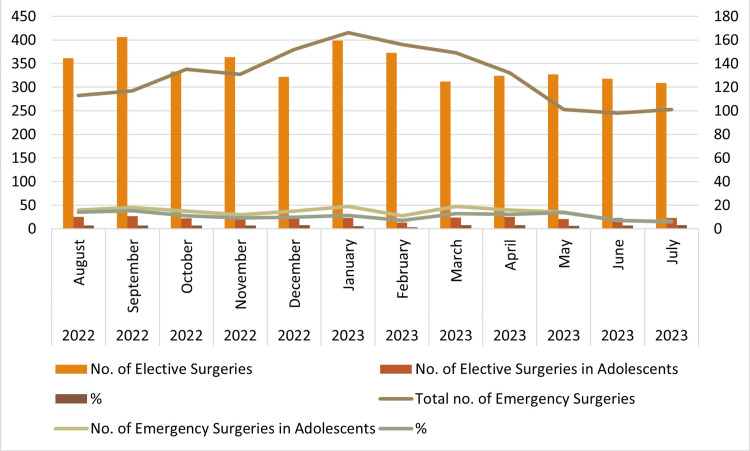
Monthly variations of surgeries

Out of 4148 elective surgeries, 277 cases (6.68%) and, among 1,551 emergency cases, 168 cases (10.83%) were in the adolescent age group. The month-wise variation of cases is enumerated in Table 1 and Figure 2.

**Table 1:** Variation of cases in different months of the year (n=5699)

Year	Month	Total no. of elective surgeries	No. of elective surgeries in adolescents	%	Total no. of emergency surgeries	No. of emergency surgeries in adolescents	%
2022	August	361	25	6.93	113	16	14.16
2022	September	406	27	6.65	117	18	15.38
2022	October	333	22	6.61	135	15	11.11
2022	November	364	26	7.14	131	12	9.16
2022	December	322	25	7.76	152	15	9.87
2023	January	399	23	5.76	166	19	11.45
2023	February	373	13	3.49	156	11	7.05
2023	March	312	24	7.69	149	19	12.75
2023	April	324	25	7.72	132	16	12.12
2023	May	327	21	6.42	101	14	13.86
2023	June	318	23	7.23	98	7	7.14
2023	July	309	23	7.44	101	6	5.94
Total		4148	277	6.68	1551	168	10.83

**Figure 3:**
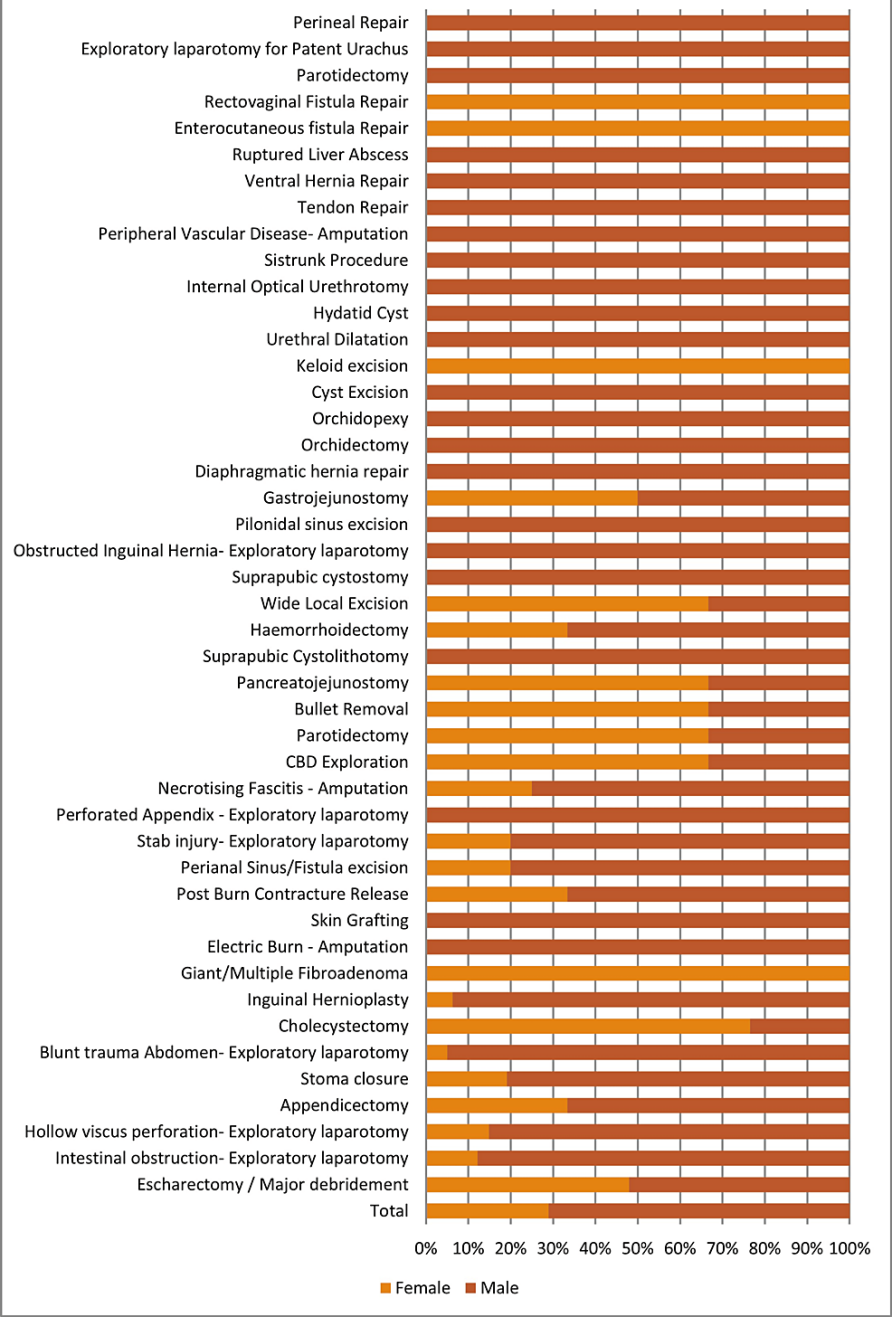
List of major surgeries with gender distribution CBD: common bile duct

The major surgeries, along with their age and sex distribution, are mentioned in Table 2 and Figure 3.

**Table 2:** List of elective and emergency major surgery done (n=315)

Name of procedure	Sex		Age								Total
	M	F	13	14	15	16	17		18	19	
Abdominal surgeries											
Exploratory laparotomy for intestinal obstruction	27	4	1	2	5	6	4	10		3	31
Appendicectomy	23	4	2	5	3	1	6	3		7	27
Exploratory laparotomy for hollow viscus perforation	20	9	4	2	4	4	7	4		4	29
Stoma closure	17	4	2	0	2	5	3	2		7	21
Exploratory laparotomy for blunt trauma to the abdomen	19	1	3	2	1	3	2	6		3	20
Cholecystectomy	4	13	1	1	2	2	5	0		6	17
Exploratory laparotomy for patent urachus	1	0	0	0	0	0	0	0		1	1
Exploratory laparotomy for stab injury	4	1	0	0	0	1	1	0		3	5
CBD exploration	1	2	0	0	0	2	0	1		0	3
Pancreatico-jejunostomy	1	2	0	0	0	2	0	0		1	3
Gastro-jejunostomy	1	1	0	1	0	0	0	0		1	2
Exploratory laparotomy for perforated appendix	5	0	1	0	1	0	0	1		2	5
Exploratory laparotomy for ruptured liver abscess	1	0	0	0	0	0	0	0		1	1
Hydatid cyst	1	0	0	0	0	0	0	1		0	1
Hernia repair surgeries											
Exploratory laparotomy for obstructed inguinal hernia	3	0	0	1	0	0	1	0		1	3
Inguinal hernioplasty	15	1	0	2	1	2	3	3		5	16
Diaphragmatic hernia repair	2	0	0	0	0	1	0	0		1	2
Ventral hernia repair	1	0	0	0	0	0	1	0		0	1
Genitourinary surgeries											
Suprapubic cystolithotomy	3	1	1	0	1	0	0	1		0	3
Orchidectomy	2	0	0	0	1	1	0	0		0	2
Suprapubic cystostomy	3	0	0	0	1	0	2	0		0	3
Orchidopexy	1	0	0	0	0	0	0	1		0	1
Urethral dilatation	1	0	0	0	0	0	0	0		1	1
Burn management											
Major debridement/escharectomy for burn injury	25	23	3	7	5	2	8	13		10	48
Amputation for electric burn	9	0	2	2	1	1	2	1		0	9
Post-burn contracture release	4	2	1	2	0	1	1	1		0	6
Amputations											
Amputation for necrotising fasciitis	3	1	1	0	1	2	0	0		0	4
Amputation for peripheral vascular disease	1	0	0	0	0	0	1	0		0	1
Perineal surgeries											
Perianal sinus/fistula excision	4	1	0	0	0	2	0	1		2	5
Haemorrhoidectomy	2	1	0	0	0	0	1	0		2	3
Pilonidal sinus excision	2	0	0	0	0	0	1	0		1	2
Perineal repair	1	0	0	0	0	0	0	1		0	1
Rectovaginal fistula repair	0	1	0	0	0	0	0	0		1	1
Other surgeries											
Wide local excision	2	2	1	1	0	1	0	1		0	4
Parotidectomy	2	2	0	0	0	0	1	1		2	4
Giant/multiple fibroadenoma	0	12	1	2	0	2	2	2		3	12
Bullet removal	1	2	0	1	0	0	1	0		1	3
Skin grafting	9	0	1	0	0	2	0	4		2	9
Enterocutaneous fistula repair	0	1	0	0	0	0	0	1		0	1
Tendon repair	1	0	1	0	0	0	0	0		0	1
Internal optical urethrotomy	1	0	0	0	0	0	0	0		1	1
Sistrunk procedure	1	0	1	0	0	0	0	0		0	1
Submental cyst excision	1	0	1	0	0	0	0	0		0	1
Total	224	91	28	31	29	43	53	59		72	315

**Figure 4:**
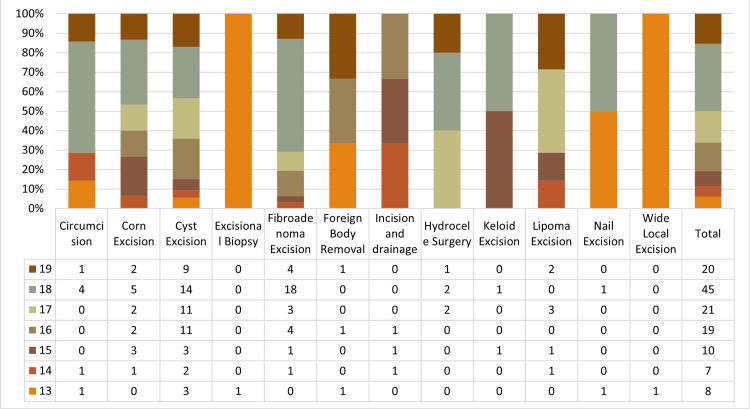
List of daycare surgeries with age distribution

The daycare surgeries, along with their age and sex distribution, are mentioned in Table 3 and Figure 4.

**Table 3:** List of daycare surgeries done (n=130)

Daycare surgery	Male	Female	Total
Cyst excision	35	18	53
Fibroadenoma excision	00	31	31
Corn excision	12	3	15
Lipoma excision	3	4	7
Circumcision	7	00	7
Hydrocele surgery	5	00	5
Foreign body removal	2	1	3
Keloid excision	0	2	3
Incision and drainage	2	1	3
Nail excision	2	1	2
Excision biopsy	1	0	1
Total	70	60	130

**Figure 5:**
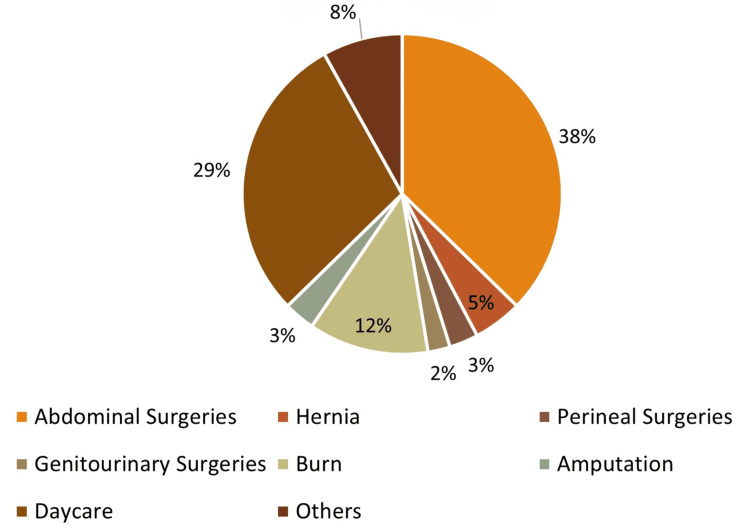
Types of surgery

Different types of surgeries were performed in the adolescent age group, of which abdominal surgeries were most common (166, 37.30%), followed by daycare surgeries (130, 29.21%; Table 4 and Figure 5).

**Table 4:** Different types of surgeries performed

Types of surgery	Number of patients	%
Abdominal surgeries	166	37.30
Hernia repair	22	4.94
Perineal surgeries	13	2.92
Genitourinary surgeries	10	2.25
Burn	54	12.13
Amputation	14	3.15
Daycare	130	29.21
Others	36	8.09
Total	445	100.00

**Figure 6:**
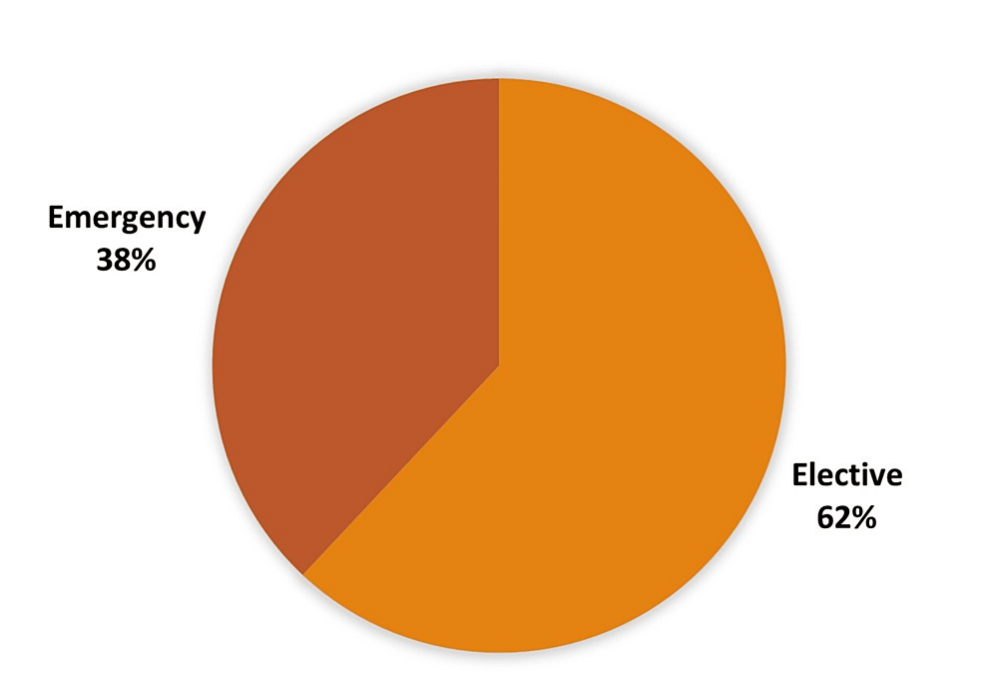
Elective vs. emergency surgery

Among 445 surgeries, 62.25% were elective surgeries, and 37.75% were emergency procedures. The distribution of emergency and elective surgeries according to sex is shown in Table 5 and Figure 6.

**Table 5:** Distribution of elective and emergency surgery among both sexes

Sex	Elective surgery	Emergency surgery	Total	p-value
Female	117	34	151	<0.001
Male	160	134	294	
Total	277 (62.25%)	168 (37.75%)	445	

**Figure 7:**
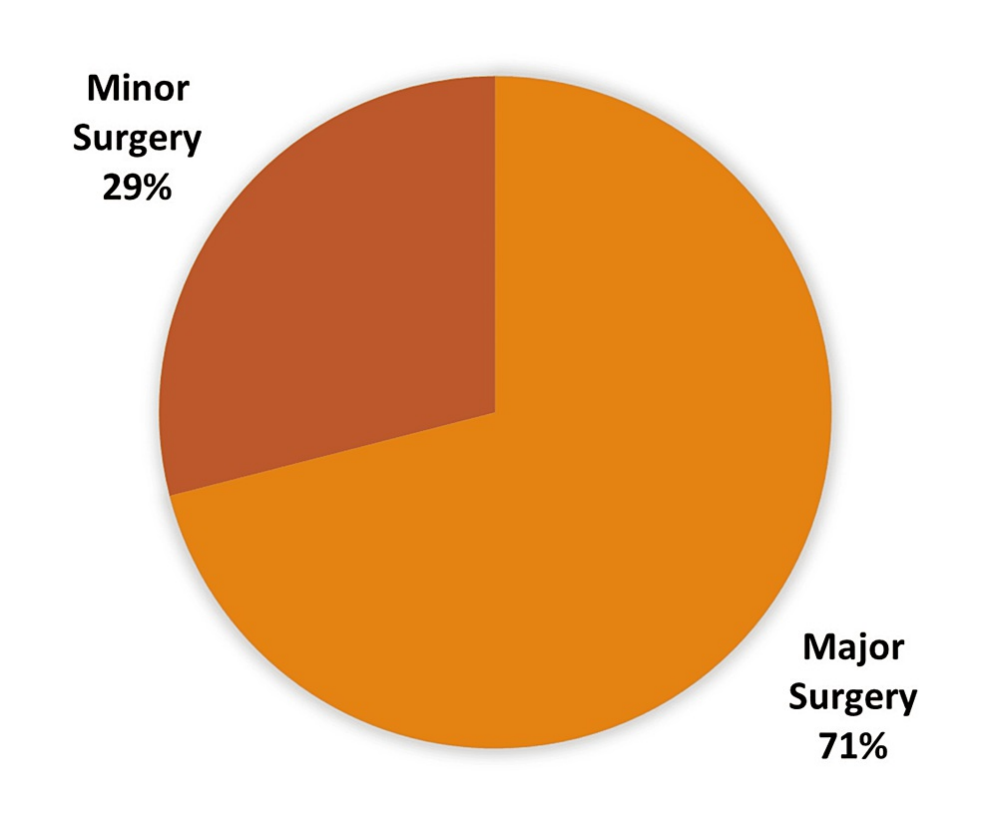
Major vs. minor surgery

Of 445 surgeries, 130 (29.21%) were minor surgeries, and 315 (70.78%) were major surgeries (Table 6, Figure 7).

**Table 6:** Type of surgery done under elective or emergency setting

Type of surgery	Elective case	Emergency case	Total
Major surgery	148	167	315
Daycare surgeries	129	1	130
Total	277	168	445

The following table shows the distribution of various major and minor surgeries in emergency as well as elective settings in different age groups (Table 7).

**Table 7:** Contingency table for age and type of surgery

Age (years)	Type of surgery	Elective cases	Emergency cases	Total
13	Major surgery	10	17	27
	Minor surgery	8	0	8
	Total	20	15	35
14	Major surgery	12	19	31
	Minor surgery	6	1	7
	Total	18	20	38
15	Major surgery	12	18	30
	Minor surgery	10	0	10
	Total	22	18	40
16	Major surgery	26	17	43
	Minor surgery	19	0	19
	Total	45	17	62
17	Major surgery	24	29	53
	Minor surgery	21	0	21
	Total	45	29	74
18	Major surgery	23	36	59
	Minor surgery	45	0	45
	Total	68	36	104
19	Major surgery	40	32	72
	Minor surgery	20	0	20
	Total	60	32	92
Total	Major surgery	149	166	315
	Minor surgery	129	1	130
	Total	277	168	445

Different anaesthesia techniques were used in major and daycare surgeries and are shown in the following table. In major surgeries, general anaesthesia (GA) was the most common technique (172/315, 54.60%), while in daycare procedures, local anaesthesia (LA) was the most common (127/130, 97.69%; Table 8).

**Table 8:** Different techniques of anaesthesia used GA: general anaesthesia, LA: local anaesthesia

Anaesthesia	Major surgery	Daycare surgery	Total
Brachial plexus block	9	0	0
GA	172	0	172
LA	54	127	181
Spinal anaesthesia	77	1	78
GA with epidural anaesthesia	2	0	2
GA with spinal anaesthesia	2	0	2
Total intravenous anaesthesia	1	0	1
Total	315	130	445

**Figure 8:**
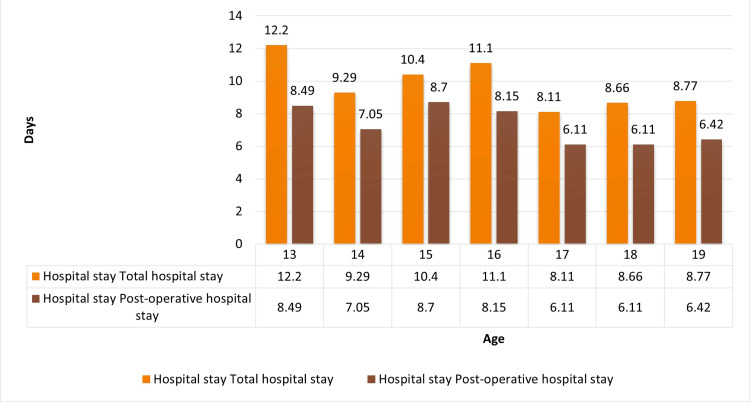
Trend of variation of hospital stay in different ages

Total and postoperative hospital stays in different age groups are shown in the following figure. Maximum days of total hospital stay were seen at age 13 years, while postoperative hospital stay was highest at 15 years (Figure 8).

Deaths among all patients included in this study were 29 (6.52%) and were recorded only after major procedures (Table 9).

**Table 9:** Outcome among the major and minor cases

Outcome	Major cases	Minor cases	Total	p-value
Death	29	0	29	<0.001
Discharged	286	130	416	
Total	315	130	445	

**Figure 9:**
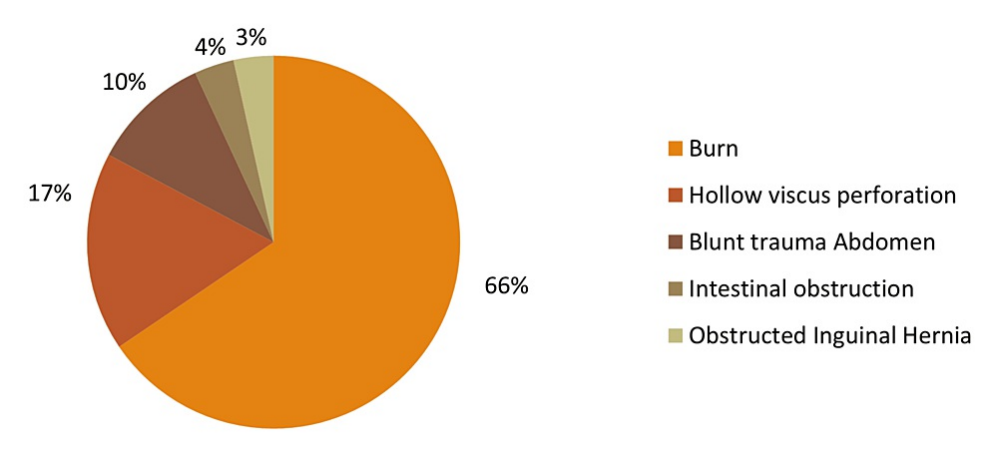
Causes of death

Various causes of death are shown in the following table. Among 29 deaths, the most common cause was burn (19, 65.52%; Table 10, Figure 9).

**Table 10:** Causes of death

Causes of death	No. of patients
Burn	19
Hollow viscus perforation	5
Blunt trauma abdomen	3
Intestinal obstruction	1
Obstructed inguinal hernia	1

## Discussion

In a one-year span, a total of 5699 surgical procedures were done, out of which 72.78% were elective and the rest were emergency procedures. In this study, 445 cases (7.80%) were in the adolescent age group. Males were 294 (66.07%), and 151 (33.93%) were females in the population studied (Figure 1).

No study is available regarding general surgical needs among the adolescent age group. Only a few studies are available on the surgical needs (general surgery, obstetrics and gynaecological, and ophthalmological surgeries, etc.) of the general population. Most studies reported that, among all surgeries, general surgery has the highest proportion.

Bhandarkar et al. (2020) [11] studied the surgical needs of the Indian general population per year for all age groups. They reported that out of the total 3646 surgeries, including general surgeries, obstetrics and gynaecological surgeries, and ophthalmological surgeries, the maximum was general surgical procedures (26%). Stokes et al. (2017) [12] in their study of the Papua New Guinea population reported that out of the total surgeries, 50% were general surgeries.

In our study, out of 445 cases, males were 294 (66.06%), while females were 151 (33.94%). Out of 315 major cases, 224 (71.11%) were males and 91 (28.89%) were females, and among 130 daycare surgeries, 70 (53.84%) were males and 60 (46.16%) were females. Among 277 elective cases, 160 (57.76%) were males and 117 (42.24%) were females. Among 168 emergency cases, most of them were males (79.76%).

In a study by Bhandarkar et al. (2020) [11], out of 8633 surgeries in the 10-19-year-old age group, 4535 (52.53%) were males and 47.47% were females. Among 1354 surgeries (general and GI surgery), 828 (61.15%) were performed on males and 527 (38.85%) were on females.

The age group of 18 years turned up the highest in both OPD and emergency departments. The mean age was 16.757 years, with the majority of the patients belonging to the late adolescent age group of 18 and 19 years (23.37% and 20.67%, respectively; Figure 1). The ratio of elective and emergency procedures in females is 33.93%, while in males it is 66.07%. This difference was found to be significant with a p<0.001 in the chi-square test.

Among the 18-year-old age group of patients, 36 emergency cases reported the most common conditions of flame burn (10), intestinal obstruction (8), and 68 OPD cases with the most frequent indication of fibroadenoma (20) and sebaceous cyst (10). While in the age group of 19 years, emergency cases were 32, with the most common cause of visit being flame burn (10), hollow viscus perforation (7), and 60 OPD cases with the most common indication of a sebaceous cyst (9), cholelithiasis (6), and fibroadenoma (6).

Out of 445 surgeries, the most common surgery was cyst excision (54, 12.13%), followed by fibroadenoma excision (43, 9.66%), and flame burn management (40, 8.99%). Hernia repair was performed in 22 patients (4.95%) of all general surgeries in the adolescent age group.

Hernia repair (133, 40.42%) was the most common general surgery, followed by abscess drainage (51, 15.50%), circumcision (45, 13.67%), and gallbladder surgery (44, 13.37%) in the series of Bhandarkar et al. (2020) [11], who studied surgical need in all age groups.

The most frequent emergency operation done, out of a total of 168, was exploratory laparotomy (87, 19.55%) and major debridement for burn (48, 10.8%), followed by appendicectomy (14) and amputation (14). The most common indication for emergency exploratory laparotomy was hollow viscus perforation (29, 33.33%), followed by intestinal obstruction (25, 28.74%) and blunt trauma abdomen (20, 22.99%). Among the 29 hollow viscus perforations, 18 were gastroduodenal, and 11 were ileal. Out of 14 amputations, nine were for electric burn injuries. Almost all emergency surgeries were major operations, except for one case of circumcision in a 14-year-old boy with phimosis (Table 2, Figure 3).

Among the remaining 277 elective cases, 129 were daycare surgeries, and 148 were major surgeries. The most common daycare procedure was cyst excision (53/129, 40.10%), followed by excision of fibroadenoma of the breast (31/129, 24.03%), and corn excision (15/129, 11.63%; Table 3, Figure 4). Among the 148 major elective cases, the most common procedure was stoma closure (21/148, 14.19%) and cholecystectomy (17/148, 11.49%), followed by inguinal hernia repair (15/148, 10.14%; Table 2).

While observing the use of the anaesthesia technique, the most common method was LA in 181 cases (40.67%), followed by GA in 172 cases (38.65%). The other methods used were spinal anaesthesia (78, 17.53%), brachial plexus block (9, 2.02%), and total intravenous anaesthesia in one case (0.22%) (Table 8). All the daycare surgeries were done under LA except one case of foreign body removal, which was done under spinal anaesthesia. Bhasin et al. (2011) [13] reported that 58% of surgeries were performed under GA, followed by LA in 32% and spinal anaesthesia in 10%.

The duration of the hospital stay was analysed. The range varied from a minimum of 0 days, i.e., same-day discharge, to a maximum of 73 days. The mean duration was 9.42 days, and the median was seven days. The mean duration of hospital stay was analysed again for different age groups, and the maximum stay was in the age group of 13 years. The postoperative hospital stay was calculated from the day of operation to the day of discharge. The mean value was 8.69 days. It was then analysed against age groups, and the maximum mean value of 13.5 days was seen in 18-year-olds (Figure 8). In the study of Bhasin et al. (2011) [13], the mean postoperative hospital stay was four to seven days in the urban population of East Delhi.

The outcome of the patients was analysed into two possibilities: discharge or death. In daycare procedures, the mortality rate was 0, while for post-major procedures, there were 29 deaths (9.02%), all after emergency procedures. The contingency table in Table 9 depicts the distribution of the outcome between major and minor cases. Among 29 deaths, the most common cause was burn (19), followed by hollow viscus perforation (5) and blunt trauma abdomen (3). Among the five deaths due to hollow viscus perforations, four were ileal perforations, and one was antral perforation (Table 10, Figure 9). The highest frequency of death was seen in the age groups of 17 and 18 years of age, which were seven each. This correlation of outcome with age was not significant (p=0.795). The distribution was almost equal in both sexes, with male mortalities at 15 (51.7%) and female mortalities at 14 (48.3%). The value was 0.092, hence not significant.

Overall mortality was 2.4% after elective surgeries, as reported by Agarwal et al. (2021) [14] in their study of postoperative outcomes following elective surgery in all age groups in India. Vester-Andersen et al. (2014) [15] reported 18.5% mortality within 30 days of emergency major GI surgeries.

## Conclusions

This study sheds light on the surgical needs and outcomes within the adolescent age group, an area often overlooked in medical research. The findings reveal a significant proportion of surgeries conducted in adolescents, with males comprising a substantial majority. The predominance of elective procedures underscores the importance of addressing adolescent health concerns proactively. The prevalence of conditions such as cyst excision and fibroadenoma excision highlights specific surgical needs within this demographic. Moreover, the study emphasises the critical role of emergency surgeries in managing acute conditions among adolescents, with exploratory laparotomy emerging as a common intervention. Understanding the distribution of surgical procedures and their outcomes in adolescents is crucial for devising targeted healthcare strategies.

While the mortality rate in elective surgeries remains relatively low, the study underscores the sobering reality of mortality associated with emergency procedures, particularly in cases like burn injuries and hollow viscus perforations. These findings underscore the need for robust emergency care protocols tailored to the unique physiological and psychological needs of adolescents. Furthermore, the analysis of anaesthesia techniques and postoperative hospital stays provides valuable insights into optimising perioperative care for adolescent patients. By considering factors such as the duration of hospitalisation and anaesthesia methods, healthcare providers can strive to enhance patient outcomes and experiences. Overall, this study underscores the importance of recognising and addressing surgical needs in the adolescent population. Further research and concerted efforts are warranted to refine surgical care protocols, improve outcomes, and ensure the holistic well-being of adolescents undergoing surgical interventions.
